# The rs3737964 single‐nucleotide polymorphism of the chloride channel‐6 gene as a risk factor for coronary heart disease

**DOI:** 10.1002/mgg3.163

**Published:** 2015-07-14

**Authors:** Li Zhang, Tao Zhang, Zhengkai Xiang, Shengqiang Lu

**Affiliations:** ^1^ Intensive Care UnitHubei Cancer HospitalWuhan430079China; ^2^ Department of UltrasoundHubei Maternal and Child Health HospitalWuhan430070China; ^3^ Department of Chest SurgeryHubei Cancer HospitalWuhan430079China

**Keywords:** Chloride channel‐6, coronary heart disease, genetic variation, single‐nucleotide polymorphism

## Abstract

The present study investigates the association of single‐nucleotide polymorphisms (SNPs) on the chloride channel‐6 (CLC‐6) gene with coronary heart disease (CHD) in China. We carried out a large case–control study among 1193 CHD patients and 1200 unrelated healthy control subjects. Information on the participants' health status was collected through the modified Inter‐heart questionnaire. Genomic DNA from peripheral blood samples was analyzed for the genotypes of rs3737964 and rs3737965 SNPs on the CLC‐6 gene using Taqman probe‐based quantitative real‐time PCR (qPCR). We compared the collected data between the case group and the control group by chi‐square test and t/nonparametric test. Furthermore, we performed logistic regression to evaluate factors associated with CHD. The frequency of *TT* genotypes in rs3737964 was significantly higher in CHD patients compared to the control group, with an odds ratio (OR) of 2.32 (95% confidence interval, CI: 1.17–4.06, *P *=* *0.016). The association of CHD with *TT* genotype was even stronger in smoking population after adjusting for confounders (OR = 3.19, 95% CI: 1.04–9.79, *P *=* *0.043). Multivariate logistic regression showed the CHD risk associated with *TT* genotype in rs3737964 was particularly among population who were more than 60 years old, smoking, and male (*P *=* *0.023, 0.008 and 0.043, respectively). The present study has revealed that rs3737964 SNP of CLC‐6 was associated with CHD. In particular, subjects with *TT* genotype who were 60‐plus years old, with smoking habit or were male were more susceptible to CHD.

## Introduction

Coronary heart disease (CHD) is the leading cause of death globally (Neubeck et al. 2009). It is a multifactorial disease caused by both genetic and environmental factors (Pranavchand and Reddy 2013). Environmental factors for CHD such as smoking, advanced age, male gender, diabetes mellitus and hypertension have been identified (Agewall 2008; Pizzi et al. 2008; Verwoert et al. 2012). Nevertheless, the underlying genetic mechanisms of CHD are much less understood and have been largely ignored. Understanding the genetic variation responsible for CHD is crucial to identify risk factors, which may enable accurate determination of an individual's risk of CHD.

Chloride channels (CLCs) are widely expressed in the cardiovascular system (Liu et al. 2007; Duan 2011). In recent years, many studies have devoted to investigate the relationship between CLCs and cardiovascular diseases. Some studies have proven that CLCs were involved in many cardiovascular diseases, such as myocardial ischemic, myocardial hypertrophy, myocardial fibrosis and oxidative damage to cardiac myocytes (Heusch et al. 2001; Duan 2009, 2011).

CLC‐6 gene, located on chromosome 1p36, was involved in cyclic adenosine monophosphate‐dependent protein kinase A signaling pathway and transmembrane transport of small molecules, which are crucial for homeostasis of cardiovascular cells (Barriere et al. 2009; Adkins and Curtis 2015). Polymorphisms of several other genes locating on chromosome 1p36, such as human B‐type natriuretic peptide precursor, natriuretic peptide A and methylene tetrahydrofolate reductase, have been shown to be associated with an increased risk of CHD. All the above evidence has triggered studies about single‐nucleotide polymorphisms (SNPs) on CCL‐6 gene. To date, few polymorphisms have been identified on CCL‐6 gene, and the association of the rs3737964 and rs3737965 SNPs with CHD is largely unexplored. Here in this study, we carried out a large case–control study in the Chinese population to determine whether the two single‐nucleotide polymorphisms, SNPs (rs3737964 and rs3737965) of CLC‐6 gene are associated with CHD and further confirm the attributable risk genotype of the selected SNPs by the method of molecular biology and statistical analysis.

## Materials and Methods

### Study population

The study population was composed of 1193 CHD and 1200 age‐ and gender‐frequency‐matched controls. Patients were recruited from three hospitals (Union Hospital, Tongji Hospital and Wugang General Hospital) in Wuhan, China. CHD was defined according to the World Health Organization criteria (World Health Organization, 1979). Participants were recruited from January, 2002 to December, 2006. The questionnaire and methods used in the survey were consistent throughout the 5‐year period. In this study, patients were considered having CHD if coronary angiogram which was performed at time of enrollment showed there was stenosis ≥50% in one or more of the major segments of coronary arteries for the first time. A total of 1193 patients diagnosed with CHD were recruited in the case group. We recruited the controls from the local inhabitants. Exclusion criteria of the control group were: having CHD, having severe liver or renal diseases, having peripheral vascular diseases and having kinship with a recruited participant. We screened participants for the above criteria by checking their medical history, and performing clinical examinations and electrocardiography.

People with hypertension, diabetes, and hyperlipidemia are prone to CHD. We defined subjects having hypertension if their systolic blood pressure was ≥140 mmHg or diastolic pressure was ≥90 mmHg or they were treated with hypotensive drugs. Subjects were considered having diabetes if they were on hypoglycemic treatment or met the 1999 World Health Organization diagnostic criteria (World Health Organization, 1979). Hyperlipidemia was defined as blood levels of cholesterol level ≥5.72 mmol/L or triglyceride ≥1.7 mmol/L. CHD family history was defined as at least one of the first‐degree family members had CHD. Those who had on average at least one cigarette per day with a continuous smoking history for more than 1 year were defined as smokers. Body mass index (BMI) is a measure of body fat and was calculated as weight in kilograms/height in m^2^.

### Data collection

The epidemiological data were collected by the Inter‐heart questionnaire (Steyn et al. 2005). Demographic characteristics collected were age and gender. General health‐related characteristics collected were disease history of hypertension, diabetes, family history of CHD. We also collected data on smoking and alcohol consumption. Systolic and diastolic blood pressure, weight, height, fasting glucose, cholesterol, and triglycerides were measured among subjects. Age, systolic and systolic blood pressure, weight, height, fasting glucose, cholesterol, and triglycerides were used as continuous variables, all other variables were categorical.

### Genotyping

Genomic DNA was extracted from venous blood of each subject with Puregene kit. To determine the gene variations in SNPs rs3737964 and 3737965, we analyzed the sequence of CLC‐6 by Taqman probe‐based qPCR analysis using the following primers:

rs3737964 sense, 5′‐TCAAATAGGAACCAGCCCTCAAAAA‐3′, and anti‐sense, 5′‐ TGTAGATCCTCACCCACATGGT‐3′, relative probe sequence, VIC‐CTCACCCCTGAAAGG and FAM‐TCACCCCCGAAAGG; rs3737965 sense,
5′‐ TGCGGGCCCAGATTGG ‐3′, and anti‐sense,
5′‐ CCCGAGGAGCTGGTAAGAAG ‐3′, relative probe sequence, VIC‐ TCTCTCAGTCCCTTAGCA and FAM‐ TCTCTCAGTCCTTTAGCA.

PCR was carried out in a 5 *μ*L reaction system in total, including the following: 10 ng DNA, 2.5 *μ*L 2 × Taqman universal PCR Master mix (no UNG), 0.125 *μ*L 40 × SNP Probe/Primer mix. Amplification of the target DNA was performed with the following condition: 95°C for 10 min, followed by 49 cycles of 92°C for 15 sec and 60°C for 1 min. Negative controls were included in each 384‐well format. Automatic allele calling with the default settings was carried out by ABI 7900HT data collection and analyzed by SDS software version 2.2.1 (SDS 2.2.1) (Zhou et al. 2008). Taqman universal PCR Master mix (no UNG) and SNP Probe/Primer mix were from Invitrogen.

### Statistical analysis

SPSS 10.0 was used to conduct the statistical analysis. We checked whether the distribution of the polymorphisms was consistent with Hardy–Weinberg equilibrium by Chi‐square test. Categorical variables were compared by Chi‐square test. Continuous variables were compared by independent Student's *t*‐test. If results from independent Student's *t‐*test showed the patient and control group do not have homogeneity of variance, nonparametric test was used to compare continuous variables. *P *<* *0.05 was considered significant. We performed univariate logistic regression analysis of CHD and possible risk factors. The variables attaining *P *<* *0.20 significance in univariate analysis were included in the multivariate regression analysis. Only variables achieving *P *<* *0.05 significance were retained in the final model using backward stepwise elimination. The degree of association was expressed as odds ratios with 95% confidence interval (CI).

## Results

### General information of the subjects

General information of CHD patients and controls was shown in Table 1. No significant difference in age and gender was observed between the CHD patients and controls (*P *= 0.061 and *P *= 0.93). Compared with the controls, the CHD patients had higher systolic blood pressure and blood sugar, and were more likely to be a smoker. The CHD patients were also more likely to smoke heavily (*P* < 0.001). Moreover, the CHD patients were more likely to have a history of hypertension and diabetes, and a family history of CHD (*P *<* *0.001). However, the CHD patients presented lower cholesterol.

**Table 1:** General characteristics of the CHD and control subjects in Wuhan, China

Category	Patients (*n* = 1193)	Controls (*n* = 1200)	*P* value
Gender (male/female)	836/357	839/361	0.93
Age (mean ± SD)	60.1 ± 10.0	59.4 ± 9.5	0.061
Systolic blopressure (mmhg)	135.2 ± 24.5	130.4 ± 20.3	<0.001
Diastolic (mmhg)	82.3 ± 14.6	82.1 ± 10.9	0.68
Body mass index (BMI)	24.5 ± 3.6	24.3 ± 3.3	0.32
Fasting glucose (mmol/L)	6.5 ± 3.1	5.2 ± 2.0	<0.001
Cholesterol (mmol/L)	4.5 ± 1.1	5.0 ± 1.3	<0.001
Triglycerides (mmol/L)	1.7 ± 1.2	1.7 ± 1.3	0.49
Smoke (no/yes)	480/679 (41.4/58.6)	603/500 (54.7/45.3)	<0.001
Cigarettes per year (pack, mean ±SD)			
0	480 (41.4)	603 (54.7)	<0.001
0–31	305 (26.3)	271 (24.6)	
31+	374 (32.3)	229 (20.8)	
Alcohol consumption (no/yes)	860/321 (72.8/27.2)	781/412 (65.5/34.5)	<0.001
Hypertension history	852 (72.0)	380 (34.8)	<0.001
Diabetes history	345 (29.3)	77 (7.1)	<0.001
Family history	172 (14.4)	38 (3.2)	<0.001

Categorical variables such as gender, smoking status, alcohol consumption, history of hypertension, history of diabetes and family history of CHD were compared by Chi‐square test. Continuous variables were compared by independent Student *t* test or nonparameter test. CHD, coronary heart disease.

### Association between the CLC‐6 SNPs and CHD

**Figure 1:**
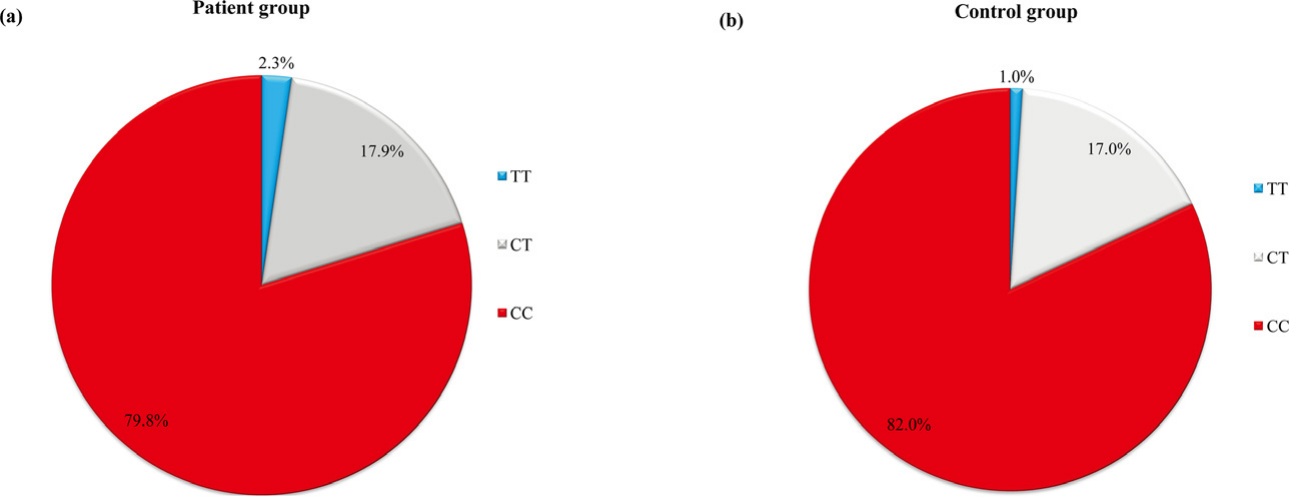
Distribution of rs3737964 geno‐types in the (A) 1184 CHD and (B) 1188 control subjects in Wuhan, China. There were 21 missing values because results of PCR of samples from nine patients and 12 controls were unreadable.

**Figure 2:**
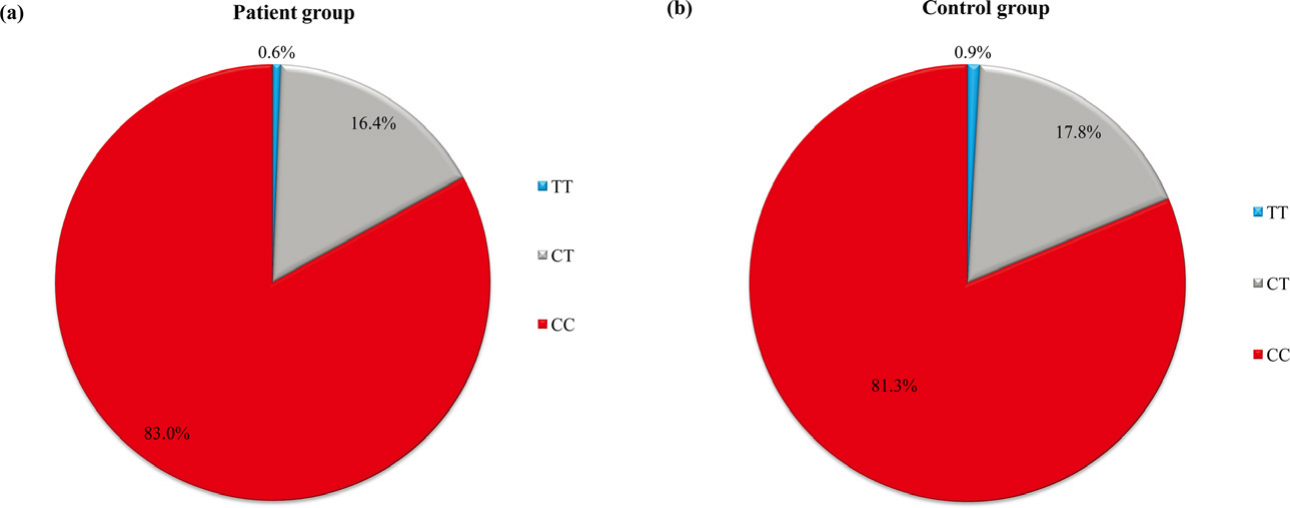
Distribution of rs3737965 geno‐types in the (A) 1190 CHD and (B) 1193 control subjects in Wuhan, China. There were nine missing values because results of PCR of samples from three patients and six controls were unreadable.

Chi‐square test showed that the distribution of SNPs rs3737964 and rs3737965 in chloride channel‐6 gene was in Hardy–Weinberg equilibrium (*χ* ^2^ = 8.86 with *P *=* *0.18 and *χ* ^2^ = 0.72 with 0.20, respectively) among CHD patients and controls. The frequencies of different genotypes of rs3737964 and rs3737965 in the CHD patients and controls were reported in Figures 1, 2. For the SNP rs3737964, the *TT* genotype in the CHD patients was significantly elevated compared to that of the controls (Fig. 1). For the SNP rs3737965, there was no significant difference between the CHD patients and the controls.

The association between rs3737964 and CHD was shown in Table 2. Compared with *CC* genotype, the *TT* genotype was significantly associated with CHD (OR = 2.32, 95% CI: 1.17–4.60, *P *=* *0.016). The association was even higher after adjusting for other confounders such as age, gender, smoking, drinking, exercise, weight, triglycerides, hypertension, diabetes and family history of CHD (adjusted OR = 2.93, 95% CI: 1.29–6.65, *P *=* *0.010).

**Table 2:** Analysis of association between rs 3737964 polymorphisms and CHD

Factor	Cases number (%)		Controls number (%)		Univariate OR (95% CI)^a^	Multivariate OR (95% CI)	*P* value
Genotype							
CC	945	79.8	974	82.0	1.00	1.00	0.010
TT	27	2.3	12	1.0	2.32 (1.10–4.60)^b^	2.93 (1.29–6.65)	
CT	212	17.9	202	17.0	1.08 (0.88–1.33)	1.16 (0.90–1.50)	
Age							
≤60	578	48.8	597	50.3	1.00	1.00	0.023
>60	606	51.2	591	49.7	2.17 (1.89–2.86)	2.06 (1.77–2.92)	
Gender							
Male	830	70.1	832	70.0	1.00	1.00	0.008
Female	354	29.9	356	30.0	0.80 (0.76–0.93)	0.64 (0.53–0.88)	
Smoking history							
No	476	41.4	594	54.4	1.00	1.00	0.043
Yes	674	58.6	497	45.6	2.13 (2.02–4.36)	2.06 (1.98–4.12)	

a Confounders such as smoking index (packs of cigarettes per year), cholesterol level, history of alcohol assumption, hypertension, and diabetes, and family history of CHD were not significant from the univariate logistic regression (*P* > 0.05) and were not included in the multivariate logistic regression. CHD, coronary heart disease.

b *P* = 0.016.

Multivariate logistic regression results adjusting for relevant confounders were shown in Table 2. The significant association between *TT* genotype and CHD was particularly found among subjects who were above 60 years old (OR = 2.06, 95% CI: 1.77–2.92, *P* = 0.023), male (OR = 3.67, 95% CI: 1.40–9.61, *P = *0.008); and smokers (OR = 2.06, 95% CI: 1.98–4.12, *P = *0.043).

## Discussion

Our large case–control study provides the first evidence that SNP rs3737964 of the chloride channel‐6 is associated with CHD. Our results showed that the *TT* genotype of the SNP rs3737964 was associated with having CHD. In logistic regression analysis, the *TT* genotype was significantly associated with CHD after adjusting for other confounders. Stratified analysis showed the significant association between *TT* genotype and CHD was particularly found among subjects who were above 60 years old, male and smokers.

Our study confirms previous epidemiological studies which suggest that the CHD patients were more likely to be a smoker, have an elevated level of blood pressure, fasting glucose, a history of hypertension, diabetes and a family history of CHD (Assmann et al. 1999a,b). The seemingly contrary result in our study that the CHD patients were more likely to have a lower level of cholesterol may due to the treatment of lipid‐lowering drugs in the CHD patients.

As a complex disease, CHD is a result of interactions of both environmental risk factors and CHD attributable genes. Identifying genetic determinants of CHD will provide somewhat accurate determination of an individual's risk of having CHD. It may also provide opportunities for population‐scale screening for CHD and new therapeutic targets of drugs (Keavney 2002). In this study, we first reported that CLC‐6 SNPs as a genetic factor for CHD, which might be used to assess an individual's risk of CHD and provide a new genetic target for treatment of CHD in the future. Given the conclusion of our large case–control study that SNP rs3737964 of CCL‐6 gene is associated with CHD, we propose that rs3737964 might be relevant with the severity of CHD and thus indicate the prognosis of CHD patients. However, since our questionnaire did not include the assessment of severity of CHD, the role of re3737964 in prediction of prognosis of CHD needs further exploration in the future. Moreover, we can screen *TT* genotype among people who are above 60 years old, male and smokers for CHD at population level to reduce the prevalence of CHD.

Chloride channel is widely expressed among cardiovascular system, including atrial cells, sinus node cells, purkinje fibers and cardiomyocytes (Kenyon and Gibbons 1979; Cheng et al. 2007; Huang et al. 2009; Wojciak‐Stothard et al. 2014). Studies reported it participates in many functional processes of cardiovascular system, including resting potential, action potential, adjusting of membrane excitability, cell permeability and volume of cells (Kunzelmann et al. 1991; Funabashi et al. 2010; Fahlke 2011; Zhu et al. 2013). These reports all suggest an important role of CLCs in normal function of cardiovascular system, which in part support our finding that SNPs in CLC‐6 is related to CHD.

At present, we have little knowledge of chloride channel‐6. Kornak et al., found that exons of chloride channel‐6 genes are very conservative among species (Kornak et al. 1999). Previous studies have shown that introns of CLC‐6 play an important role in CLC‐6 gene expression and relevant physiological processes (Eggermont 1998; Kornak et al. 1999). In our study, the two SNPs rs3737964 and rs3737965 were both sited in the intron of CLC‐6 gene, which suggests that CLC‐6 contributes to the etiology of CHD. We recommend future studies to take CLC‐6 genes into account when investigating genetic factors of CHD.

As genetic variants can be influenced by many factors, such as location, time, and nations (In, 2006). More studies need to be performed to further understand the function of SNPs in CLC‐6 in CHD patients among different populations, races and countries to get a comprehensive picture of the role of SNPs in CHD.

In summary, our study, to our knowledge, the first time reveals an association between SNP rs3737964 of the chloride channel‐6 and CHD. Our results provide a novel factor that can be used to determine an individual's risk of CHD, screen population at large and provide new therapeutic targets of drugs.

## Conflict of Interest

None declared.
